# Preparing Students to Interact With Persons With Dementia in a Research Context

**DOI:** 10.1093/geroni/igaa057.007

**Published:** 2020-12-16

**Authors:** Roberta Peterson, Elizabeth Avent, Zachary Gassoumis, Jeanine Yonashiro Cho, Bonnie Olsen

**Affiliations:** 1 Keck School of Medicine of USC, Los Angeles, California, United States; 2 University of Southern California, Los Angeles, California, United States

## Abstract

By U.S. Centers for Disease Control and Prevention estimates, the number of older adults with Alzheimer’s Disease and related dementias (ADRD) is expected to increase 278% by 2060 to affect approximately 13.9 million individuals. Research is needed to not only improve understanding and treatment of ADRD but to also study its effect on the physical, emotional, and psychological well-being of persons with dementia (PWD) and their care partners (CP). However, due to the diminishing cognitive and functional capacities of PWDs associated with the progression of ADRD over time, research efforts are sometimes hampered by a plethora of potential scientific, logistical, ethical, and emotional barriers. This session will introduce an educational approach used to train students who are interested in conducting in-home research among PWDs and their CPs and share lessons learned through the program’s pilot training of undergraduate and graduate students (N=6). Through didactic training, role-playing exercises, and experiential learning processes, trainees are equipped to accompany research project interviewers into the homes of PWD and assist in implementing research protocols. Students receive extensive training in the disease trajectories of ADRD, the impacts of disease on PWDs and their CPs, ways to communicate and interact with PWDs, best practices in promoting the protection and autonomy of human subjects with dementia, and approaches to obtaining quality data for research.

